# Transition patterns of metabolic dysfunction-associated fatty liver disease status in relation to arterial stiffness progression: a health check-up cohort study

**DOI:** 10.1038/s41598-023-35733-0

**Published:** 2023-06-15

**Authors:** Lei Liu, Changfa Wang, Shuwen Deng, Ting Yuan, Xiaoling Zhu, Yuling Deng, Yuexiang Qin, Yaqin Wang, Pingting Yang

**Affiliations:** 1 Health Management Center, The Third Xiangya Hospital, Central South University, No.138 Tongzipo Road, Yuelu District, Changsha, 410013 Hunan China; 2 General Surgery Department, The Third Xiangya Hospital, Central South University, No.138 Tongzipo Road, Yuelu District, Changsha, 410013 Hunan China

**Keywords:** Cardiovascular diseases, Metabolic disorders, Hepatology, Risk factors

## Abstract

Metabolic dysfunction-associated fatty liver disease (MAFLD) is a new diagnostic criterion based on hepatic steatosis and metabolic dysfunction. However, a comprehensive evaluation of the association of MAFLD dynamic transitions with arterial stiffness progression has yet to be conducted. This cohort study included 8807 Chinese health check-up participants (median follow-up = 50.2 months). Participants were categorized into four groups according to MAFLD status at baseline and follow-up (none, persistent, developed and regressed). Arterial stiffness progression was assessed by the annual brachial-ankle pulse wave velocity (ba-PWV) increase and arterial stiffness incidence. Compared with the non-MAFLD group, the annual increase in ba-PWV was highest in the persistent-MAFLD group [6.75 cm/s/year, (95% CI 4.03–9.33)], followed by the developed—[6.35 cm/s/year, (95% CI 3.80–8.91)] and the regressed—[1.27 cm/s/year, (95% CI − 2.18 to 4.72)] MAFLD groups. Similarly, compared with the non-MAFLD group, the persistent-MAFLD group had a 1.31-fold increased arterial stiffness risk [OR 1.31; 95% CI 1.03–1.66]. The associations of MAFLD transition patterns with arterial stiffness incidence did not differ across any clinically specific subgroups evaluated. Furthermore, the potential effect of dynamic changes in cardiometabolic risk factors on arterial stiffness incidence among persistent-MAFLD participants was mostly driven by annual fasting glucose and triglyceride increases. In conclusion, persistent MAFLD was associated with an increased risk of arterial stiffness development. Moreover, in persistent-MAFLD subjects, elevated blood glucose and triglyceride levels might facilitate the arterial stiffness incidence.

## Introduction

Nonalcoholic fatty liver disease (NAFLD) is one of the most common chronic liver diseases in modern society and is characterized by the increasing accumulation of liver fat in the absence of excess alcohol intake and other secondary causes of liver disease^1^. The prevalence of NAFLD has been reported to be 25.2% globally and 29.2% in China, and NAFLD is associated with metabolic syndrome, diabetes, and cardiovascular disease (CVD) morbidity and mortality^2–4^. While it is well understood that cardiometabolic abnormality is the hallmark disease characteristic, the definition of NAFLD lacks a unified set of “positive” criteria accounting for the key metabolic features, leading to heterogeneous disease characterization and endpoint prognostication^5^. In 2020, a panel of international experts proposed a new term, metabolic dysfunction-associated fatty liver disease (MAFLD), with broader diagnostic criteria that require the presence of metabolic risk factors in the setting of hepatic steatosis; the criteria include individuals with other concomitant liver diseases and exclude those with hepatic steatosis who do not fulfill the metabolic risk criteria^6–8^. Two recent studies reported that the prevalence of MAFLD was 34.8% among US adults and 31.5% among Chinese adults^9,10^. Studies have confirmed that the MAFLD definition might outperform its predecessor in identifying high-risk patients among those with fatty liver disease (FLD), especially in predicting the risk of cardiovascular and cerebrovascular diseases^11–14^.

At present, carotid artery intima-media thickness (CIMT), arterial stiffness (AS), coronary artery calcification (CAC) and brachial arterial flow-mediated dilation (FMD) are noninvasive techniques that generally serve as surrogate markers for subclinical atherosclerosis. They are used during the initial assessment of potential cardiovascular events and risk stratification to determine appropriate therapeutic strategies for patients with latent CVD^15^. Considerable previous studies have shown significant associations of NAFLD with subclinical atherosclerosis^15–17^. Recently, some scholars have focused on the relationship between MAFLD and CVD or subclinical atherosclerosis^18–22^. However, most studies have focused on the relationship between MAFLD and CVD morbidity and mortality rather than early-stage “subclinical atherosclerosis”. Furthermore, most of the studies have been cross-sectional with single-point assessments, which did not allow for the assessment of MAFLD dynamic changes and their contributions to subclinical atherosclerosis. Whether the subtypes of metabolic factors may modify the effect on the development of subclinical atherosclerosis remains unknown.

Therefore, assessing the progression of arterial stiffness and its association with MAFLD may help to improve our understanding of cardiovascular risks in fatty liver clinical entities. Our aim was to explore whether dynamic changes in MAFLD status were associated with the progression of arterial stiffness in a large-scale health check-up cohort. Additionally, we explored the impacts of different cardiometabolic risk factor changes on the incidence of arterial stiffness.

## Materials and methods

### Study design and participants

**Figure 1:**
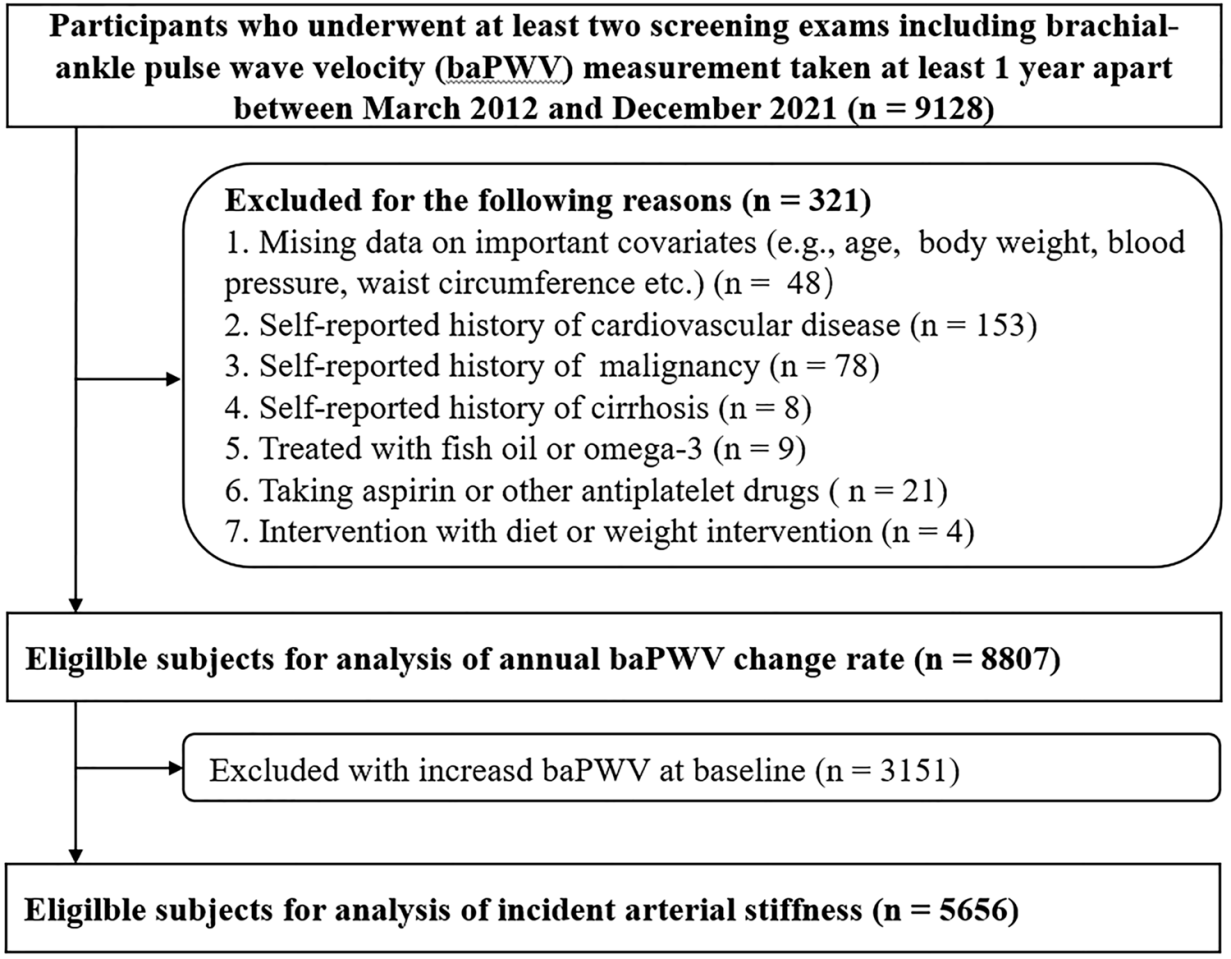
Flow chart of participant selection.

This cohort study was conducted from March 2012 to December 2021 and included healthy participants who underwent a routine health check-up examination at the Health Management Center in the Third Xiangya Hospital of Central South University (Changsha), the largest medical institution in central China. The study population consisted of subjects who underwent at least two routine health check-ups, including brachial-ankle pulse wave velocity (ba-PWV) measurement, performed at least 1 year apart (n = 9128). For the subjects (3.5%) who completed the check-up visits three times or more, we chose the measurements with the longest duration between baseline and follow-up in our analyses. We excluded 321 participants for the following reasons: missing data describing important covariates; self-reported history of malignancy, cardiovascular disease or cirrhosis; use of aspirin, other antiplatelet drugs, fish oil or omega-3 supplements; and diet or weight-loss interventions. Finally, 8807 participants were included in the cohort study population (Fig. 1). This study complied with the Declaration of Helsinki. The Institutional Review Board (IRB) of the Third Xiangya Hospital, Central South University (No. 2013BAI04B01) approved the study.

### Assessment of CVD and metabolism-related risk factors

Questionnaire-based lifestyle information and clinical and biochemical measurements were collected and quality controlled according to standard protocols and regulations. Self-report questionnaires administered via a website (https://new.selfhealth.com.cn/#/login) were used to collect data on education, current medication, previous medical diagnoses, exercise, smoking history, and alcohol consumption. Definitions of lifestyle characteristics, hypertension, type 2 diabetes and dyslipidemia are detailed in the Online Appendix. Height and body weight, waist circumference (WC) and blood pressure were measured as previously described^23^. Body mass index (BMI) was calculated as weight in kilograms divided by the square of height in meters (kg/m^2^).

Venous blood samples were drawn after an overnight fast and immediately analyzed at the clinical laboratory of Third Xiangya Hospital. Serum lipids, serum creatinine (SCr), fasting plasma glucose (FPG), alanine aminotransferase (ALT), aspartate aminotransferase (AST), albumin, globulin and total bilirubin were measured as previously described^24^. FIB-4 was used as a noninvasive marker of nonalcoholic steatohepatitis (NASH) based on the following formula: FIB-4 index = age × AST (U/L)/platelet count (× 10^9^/L)√ALT(U/L)^25^. A high FIB-4 score (≥ 1.30) is a strong predictor of the presence of liver fibrosis^26^. The estimated glomerular filtration rate (eGFR) was used as an index of renal disease based on the Modification of Diet in Renal Disease formula for Chinese subjects: eGFR = 175 × SCr^– 1.234^ × age^– 0.179^ [if female, × 0.79]^27^.

### Ascertainment of MAFLD and transition patterns

MAFLD was defined as the presence of hepatic steatosis with 1 or more of the following: (1) overweight or obesity (body mass index ≥ 23 kg/m^2^; based on the Asia–Pacific criteria); (2) type 2 diabetes; or (3) at least 2 metabolic abnormalities^7^. Metabolic abnormalities were defined as the presence of at least 2 metabolic risk abnormalities: (1) WC greater than or equal to 90/80 cm in Asian men and women; (2) blood pressure greater than or equal to 130/85 mm Hg or specific drug treatment; (3) plasma triglycerides greater than or equal to 1.70 mmol/L or specific drug treatment; (4) plasma high-density lipoprotein-cholesterol (HDL-C) less than 1.0 mmol/L for men and less than 1.3 mmol/L for women or specific drug treatment; and (5) prediabetes (FBG levels 5.6–6.9 mmol/L). Data on plasma high-sensitivity C-reactive protein (CRP) levels and homeostasis model assessment of insulin resistance scores were unavailable in our study. Fatty liver was detected by hepatic ultrasound (Logiq 9, GE Medical System, Milwaukee, WI, USA) as previously described and detailed in the Online Appendix^24^. The diagnosis of fatty liver was based on standard criteria, including parenchymal brightness, liver-to-kidney contrast, deep beam attenuation, bright vessel walls and gallbladder definition. Subjects with at least two increases in echogenicity of the liver were diagnosed with hepatic steatosis.

We assessed MAFLD status at baseline and at the end of follow-up to create the following four transition phenotypes: (1) none, those without MAFLD both at baseline and follow-up; (2) developed, those without MAFLD at baseline but with MAFLD at follow-up; (3) regressed, those with MAFLD at baseline but without MAFLD at follow-up; and (4) persistent, those with MAFLD both at baseline and follow-up.

### Assessment of arterial stiffness progression

Ba-PWV was assessed using a noninvasive, automatic oscillometric device (BP-203 RPEIII, Omron Health Medical, Dalian, China). After the subject had rested for at least 5 min in the supine position, 4 cuffs were wrapped around the upper arms and the ankles and were connected to a plethysmographic sensor (volume pulse form) and oscillometric pressure sensor. Pressure waveforms were recorded at both the brachial and tibial arteries to assess the transmission time between the initial rises in these waves. The distance between the brachial plexus and ankle was automatically calculated according to the subject’s height. Distance-transmission time analysis between the brachial plexus and volume waveforms at both ankles was used to calculate ba-PWV. Measurements were performed twice, and the mean value of the left and right sides was used in the analysis. Two trained technicians performed all measurements; the between-visit coefficient of variation (CV) of the measurements was 4.9%, and the intra-observer CV was 7.8%, as previously reported^23^.

We created two evaluation metrics to assess arterial stiffness progression. First, the annual ba-PWV increase was calculated by the difference in ba-PWV measurements (cm/s) between two visits (follow-up ba-PWV/baseline ba-PWV) divided by the time interval (years). Second, incident arterial stiffness was defined as a ba-PVW value less than 1400 cm/s at baseline but more than 1400 cm/s at the end of the visit^28^.

### Statistical analysis

Baseline characteristics are presented as the mean ± SD or median (first quartile, third quartile) for continuous variables and as percentages for categorical variables. Bonferroni correction was used for continuous variables, including *p* for trend values for the general comparison of groups and specific *p* values when comparisons were conducted with the reference group. The chi-square test was used for categorical variables.

Linear regression analysis was used to evaluate the association between the annual ba-PWV increase and MAFLD transition patterns, with the annual ba-PWV increase as the dependent variable. We followed a 2-step approach for the inclusion of covariates in the models. First, clinical covariables were entered into the univariate linear regression analysis. Those variables that showed a *p* < 0.10 in the univariate analyses or were selected a priori based on possible associations with MAFLD and atherosclerosis were entered into the multivariable regression analyses. Second, in cases of multicollinearity, waist circumference was selected instead of BMI, and systolic blood pressure was selected instead of diastolic blood pressure.

Cox regression analysis was fitted to explore the relationship between the incidence of arterial stiffness and MAFLD transition patterns in the population free of arterial stiffness at baseline. Moreover, we used Cox regression analyses to examine the relationship between the incidence of arterial stiffness and MAFLD transition patterns in specified subgroups. Additionally, analyses of cardiometabolic risk factor change rate and baseline fibrosis probability with the incidence of arterial stiffness were repeated using a restricted sample of participants with persistent MAFLD. Finally, we performed additional sensitivity analysis with the higher ba-PWV values for the right and left ankles instead of the mean values of the two sides. The regression model and covariates were the same as those described above.

All statistical analyses were performed with SPSS software version 23.0 (IBM, Armonk, New York). Differences were considered statistically significant at *p* < 0.05. The graphs were created in GraphPad Prism version 9.00 (GraphPad Software, La Jolla, California).

### Institutional review board

This study complied with the Declaration of Helsinki. The Institutional Review Board (IRB) of the Third Xiangya Hospital, Central South University (No. 2013BAI04B01) approved the study.

### Informed consent

Informed consent was obtained from all subjects involved in the study.

## Results

### Clinical profile and arterial stiffness at baseline

The overall study population comprised 8807 individuals (mean 44.6 years of age; 69.2% male). Baseline characteristics according to MAFLD progression are presented in Table 1. A higher proportion of males, current smokers and current drinkers; higher levels of metabolism-related risk factors, liver enzymes and ba-PWV; a higher prevalence of diabetes, hypertension, dyslipidemia; and increased ba-PWV value were found in participants with persistent, regressed or developed MAFLD than in those without MAFLD. Compared with the persistent MAFLD group, the regressed group showed a relatively better metabolic profile, with lower levels of BMI, WC, and triglycerides and a lower prevalence of dyslipidemia. Similar findings were observed in the population with no MAFLD compared with the population with MAFLD at baseline (Supplemental Table S1).

**Table 1:** Study population and clinical characteristics at baseline (n = 8807) stratified by MAFLD progression status.

Clinical characteristics	Total	MAFLD progression status				*P* Value^a^
		None	Developed	Regressed	Persistent	
Prevalence, n (%)	8807 (100)	3674 (41.7)	1599 (18.2)	592 (6.7)	2942 (33.4)	
Demographic factors						
Age, years	44.6 ± 9.7	46.3 ± 9.7	47.3 ± 9.6^b,c^	48.8 ± 9.7^b,c^	46.4 ± 9.1	< 0.001
Male sex, n (%)	6092 (69.2)	1897 (51.6)^c^	1170 (73.2)^b,c^	473 (79.9)^b,c^	2552 (86.7)^b^	< 0.001
University degree, n (%)	4124 (46.8)	1678 (45.7)	722 (45.2)	319 (53.9)^b,c^	1405 (47.8)	< 0.001
Lifestyle status						
Current smoker, n (%)	1933 (21.9)	627 (17.1)^c^	398 (24.9)^b^	115 (19.4)^c^	793 (27.0)^b^	< 0.001
Current drinker, n (%)	2749 (31.2)	916 (24.9)^c^	576 (36.0)^b^	175 (29.6)^b,c^	1082 (36.8)^b^	< 0.001
Physical activity, n (%)	3322 (37.7)	1567 (42.7)^c^	552 (34.5)^b^	211 (35.6)^b^	992 (33.7)^b^	< 0.001
Classic vascular risk factors						
BMI, kg/m^2^	24.7 ± 3.1	22.5 ± 2.5^c^	25.1 ± 2.7^b,c^	25.7 ± 2.2^b,c^	26.8 ± 2.5^b^	< 0.001
Waist circumference, cm	84.3 ± 9.4	77.8 ± 7.8 ^c^	85.8 ± 7.5^b,c^	87.7 ± 6.7^b,c^	91.0 ± 6.9^b^	< 0.001
Heart rate, beats/min	71.7 ± 10.9	70.9 ± 10.8^c^	71.8 ± 11.3	71.9 ± 11.1	72.6 ± 10.6^b^	< 0.001
Systolic blood pressure, mm Hg	124.3 ± 15.9	120.4 ± 16.1^c^	126.7 ± 16.3^b^	126.6 ± 14.4^b^	127.6 ± 14.7^b^	< 0.001
Diastolic blood pressure, mm Hg	78.2 ± 11.5	74.6 ± 11.0^c^	79.5 ± 11.3^b,c^	80.5 ± 11.2^b^	81.5 ± 11.0^b^	< 0.001
Hypertension, n (%)	1614 (18.3)	415 (11.3)^c^	352 (22.0)^b^	133 (22.5)^b^	714 (24.3)^b^	< 0.001
Anti-hypertensive medication, n (%)	671 (7.6)	154 (4.2)^c^	134 (8.4)^b,c^	56 (9.5)^b^	327 (11.1)^b^	< 0.001
Fasting glucose, mmol/L	5.45 ± 1.25	5.22 ± 0.97^c^	5.53 ± 1.45^b^	5.68 ± 1.48^b^	5.65 ± 1.33^b^	< 0.001
Diabetes mellitus, n (%)	646 (7.3)	139 (3.8)^c^	115 (7.2)^b,c^	75 (12.7)^b^	317 (10.8)^b^	< 0.001
Anti-diabetes medication, n (%)	288 (3.3)	64 (1.7)^c^	28 (1.8)^c^	30 (5.1)^b^	166 (5.6)^b^	< 0.001
Triglycerides, mmol/L	1.52 (1.04, 2.30)	1.13 (0.84, 1.59)^c^	1.65 (1.19, 2.40)^b,c^	1.80 (1.22, 2.61)^b,c^	2.06 (1.47, 3.11)^b^	< 0.001
HDL–cholesterol, mmol/L	1.42 ± 0.36	1.58 ± 0.38^c^	1.37 ± 0.34^b,c^	1.35 ± 0.29^b,c^	1.28 ± 0.27^b^	< 0.001
LDL–cholesterol, mmol/L	2.77 ± 0.85	2.75 ± 0.82	2.82 ± 0.85^b^	2.82 ± 0.85^b^	2.76 ± 0.89	0.012
Dyslipidemia, n (%)	3105 (35.3)	715 (19.5)^c^	619 (38.7)^b,c^	237 (40.0)^b,c^	1534 (52.1)^b^	< 0.001
Anti-dyslipidemia medication, n (%)	147 (1.7)	43 (1.2)^c^	43 (2.7)^b^	7 (1.2)	54 (1.8)^b^	0.001
Emerging risk factors and others						
Albumin, g/L	46.4 ± 2.7	46.0 ± 2.7 ^c^	46.5 ± 2.7^b,c^	46.2 ± 2.8^c^	46.8 ± 2.6^b^	< 0.001
Total bilirubin, μmol/L	15.4 ± 5.2	15.6 ± 5.1^c^	15.4 ± 5.0	15.6 ± 5.9	15.2 ± 5.1^b^	0.104
ALT, U/L	24.0 (17.0, 34.0)	19.0 (14.0, 26.0)^c^	24.0 (18.0, 34.0)^b,c^	25.0 (19.0, 35.0)^b,c^	31.0 (22.0, 45.0)^b^	< 0.001
AST, U/L	22.0 (19.0, 26.0)	21.0 (18.0, 25.0)^c^	22.0 (19.0, 25.0)^b,c^	23.0 (20.0, 26.0)^b,c^	24.0 (19.0, 21.0)^b^	< 0.001
FIB–4	0.42 (0.29, 0.60)	0.49 (0.36, 0.69)^c^	0.41 (0.29, 0.58)^b,c^	0.43 (0.30, 0.61)^b,c^	0.35 (0.24, 0.48)^b^	< 0.001
eGFR, mL/min/1.73 m^2^	108.0 (94.1, 123.9)	110.8 (96.2, 128.0)^c^	107.8 (93.7, 122.0)^b,c^	106.0 (92.7, 122.1)^b^	105.0 (92.3, 120.0)^b^	< 0.001
Arterial stiffness						
baPWV, cm/s	1326 (1206, 1477)	1280 (1166, 1424)^c^	1344 (1288, 1503)^b^	1361 (1243, 1507)^b^	1367 (1245, 1511)^b^	< 0.001
Increased baPWV, n (%)	3151 (35.8)	1032 (28.1)^c^	624 (39.0)^b,c^	250 (42.2)^b^	1245 (42.3)^b^	< 0.001
Follow-up time, year	4.18 ± 2.55	3.86 ± 2.54^c^	4.27 ± 2.47^b^	4.53 ± 2.65^b^	4.47 ± 2.54^b^	< 0.001

Values are n (%), mean ± SD, or median (first quartile, third quartile). ^a^ *p* values for continuous variables in this column reflect p for trend. ^b^ *p* < 0.05 vs. None (reference group). ^c^ *p* < 0.05 vs. persistent (reference group).

*HDL* high-density lipoprotein, *LDL* low-density lipoprotein, *ALT* alanine aminotransferase, *AST* aspartate aminotransferase, *eGFR* estimated glomerular filtration rate, *baPWV* brachial–ankle pulse wave velocity.

### MAFLD and arterial stiffness progression

**Figure 2:**
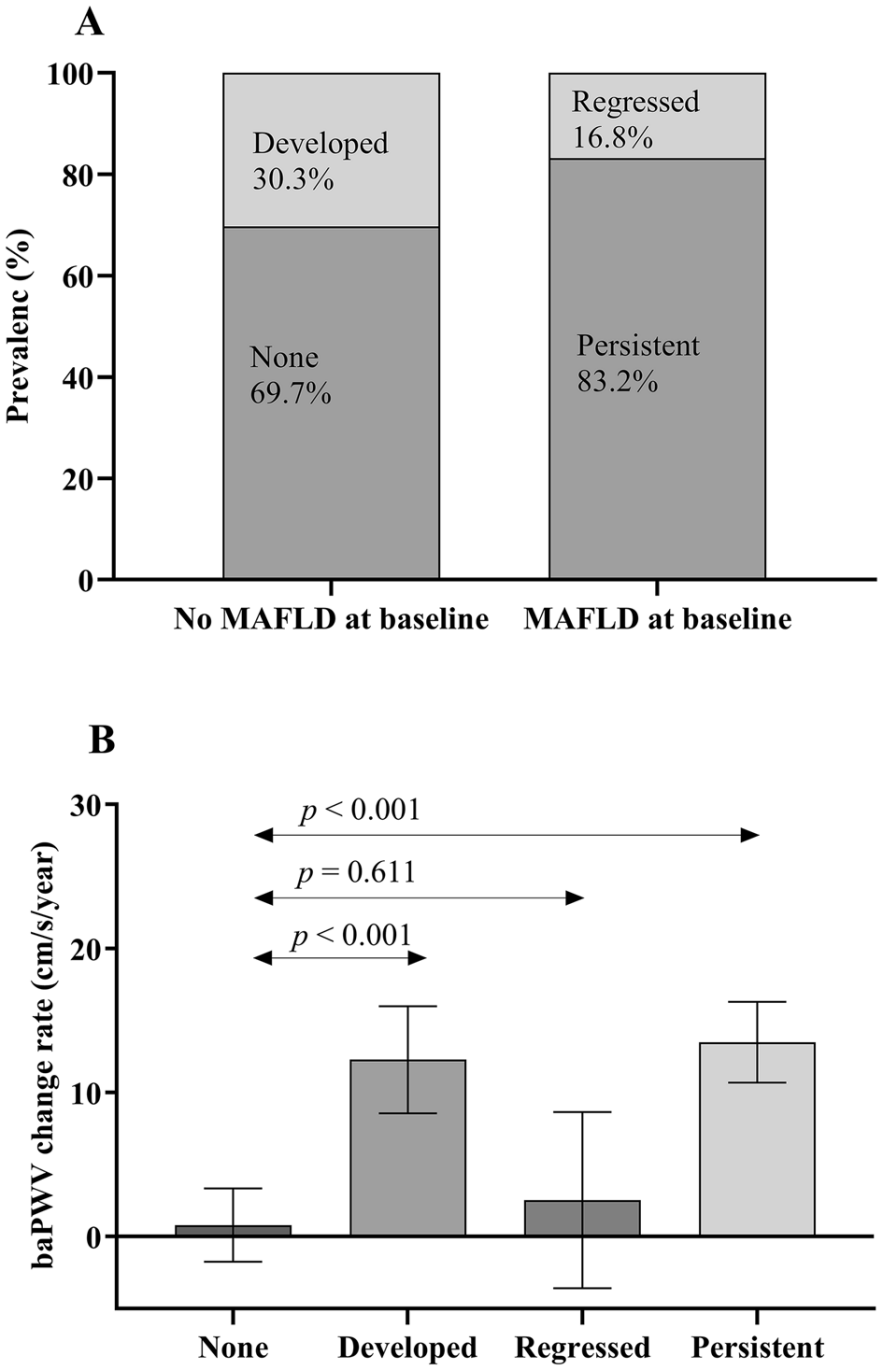
Prevalence of MAFLD status and annual ba-PWV change across MAFLD progression categories over follow-up. (**A**) Values shown as percentages (%). The distribution of MAFLD status both at baseline and follow-up. (**B**) The values shown in each column represent the mean (95% CI) ba-PWV change rate (cm/s/year) adjusted for age, sex and ba-PWV at baseline. The *P* values were assessed by analysis of covariance (ANCOVA) and Bonferroni’s tests for multiple comparisons.

The proportion of participants with MAFLD was 40.1% at baseline. Over a median of 50.2 months of follow-up, 83.2% of MAFLD patients at baseline had the same MAFLD phenotype, whereas 16.8% regressed to no MAFLD at the follow-up check-up, and 30.3% of non-MAFLD patients at baseline progressed to MAFLD at the follow-up visit (Fig. 2A). Participants in persistent and developed MAFLD categories showed a significantly increased ba-PWV change rate from baseline compared with that of the ‘none’ and ‘regressed’ MAFLD categories (Fig. 2B).

### Association of MAFLD transition patterns and the annual increase in ba-PWV

To analyze the significance of the annual increase in ba-PWV in regard to MAFLD change patterns, multivariate linear regression analyses were performed in all samples (n = 8807) (Table 2). After multivariable adjustment (Model 2), the persistent and developed MAFLD groups showed significantly greater annual increased ba-PWV [β (95% confidence interval (CI)) 9.30 (3.89–14.70) cm/s/year; β (95% (CI) 6.77 (1.22–12.33) cm/s/year, respectively] than the non-MAFLD group. The associations were attenuated but remained significant even after further adjustment for ba-PWV at baseline (Model 3). In contrast, the participants in the regressed MAFLD group were not significantly associated with an annual increase in ba-PWV.

**Table 2:** Association between MAFLD change patterns and the annual increase in ba-PWV in all population samples (n = 8807).

	The annual baPWV change rate					
	Model 1		Model 2		Model 3	
	Coefficient β (95% CI)	*P* value	Coefficient β (95% CI)	*P* value	Coefficient β (95% CI)	*P* value
MAFLD progression status						
None (n = 3674)	1.00 (Reference)		1.00 (Reference)		1.00 (Reference)	
Developed (n = 1599)	6.26 (1.50–11.02)	0.010	6.77 (1.22–12.33)	0.017	6.35 (3.80–8.91)	< 0.001
Regressed (n = 592)	− 2.16 (− 9.18 to 4.87)	0.547	− 0.20 (− 7.71 to 7.31)	0.959	1.27 (− 2.18 to 4.72)	0.471
Persistent (n = 2942)	6.34 (2.24–10.43)	0.002	9.30 (3.89–14.70)	0.001	6.75 (4.03–9.33)	< 0.001

Estimated from linear mixed models.

Model 1 adjusted for age and sex.

Model 2 adjusted for Model 1 variables plus year of screening exam, education level, current smoking, current drinking, physical activity, anti-hypertensive medication, anti-diabetes mellitus medication, lipid-lowing medication, waist circumference, systolic blood pressure, fasting glucose, triglycerides, LDL cholesterol, HDL cholesterol, FIB–4, estimated GFR, total bilirubin and albumin at baseline.

Model 3 adjusted for Model 2 variables plus baPWV at baseline.

*MAFLD* metabolic dysfunction–associated fatty liver disease, *CI* confidence interval.

### Association of MAFLD transition patterns and the incident arterial stiffness

To analyze the significance of incident arterial stiffness in regard to MAFLD transition patterns, multivariate Cox regression analyses were performed among participants without arterial stiffness at baseline (n = 5656) (Table 3). Over a median follow-up of 4.33 years, 1254 participants developed arterial stiffness. Compared with participants with non-MAFLD, participants with regressed MAFLD and developed MAFLD were not significantly associated with the risk of incident arterial stiffness in the fully adjusted models. Participants with persistent MAFLD had a 1.31-fold increased risk of incident arterial stiffness (OR 1.31; 95% CI 1.03–1.66) after full adjustment. The stratified analyses revealed that the association of incident arterial stiffness with MAFLD transition patterns was affected by baseline age, sex, diabetes, hypertension and dyslipidemia (Table 4).

**Table 3:** Association between MAFLD transition patterns and incident arterial stiffness in the population free of arterial stiffness at baseline (n = 5656).

	Person-years	Incident cases	Rate*	The incident arterial stiffness					
				Model 1		Model 2		Model 3	
				HR (95% CI)	*P* value	HR (95% CI)	*P* value	HR (95% CI)	*P* value
None (n = 2642)	10574.2	401	37.9	1.00 (Reference)		1.00 (Reference)		1.00 (Reference)	
Developed (n = 975)	4436.6	280	63.1	1.49 (1.28–1.75)	< 0.001	1.21 (0.97–1.51)	0.098	1.16 (0.93–1.45)	0.183
Regressed (n = 342)	1591.8	75	47.1	0.84 (0.65–1.08)	0.163	0.84 (0.61–1.17)	0.312	0.90 (0.65–1.24)	0.521
Persistent (n = 1697)	7519.9	498	66.2	1.54 (1.34–1.78)	< 0.001	1.41 (1.11–1.78)	0.005	1.31 (1.03–1.66)	0.025

Estimated from Cox proportional hazard models.

Model 1 adjusted for age and sex.

Model 2 adjusted for Model 1 variables plus year of screening exam, education level, current smoking, current drinking, physical activity, anti-hypertensive medication, anti-diabetes mellitus medication, anti-dyslipidemic medication, waist circumference, systolic blood pressure, fasting glucose, triglycerides, LDL cholesterol, HDL cholesterol, FIB–4, estimated GFR, total bilirubin and albumin at baseline.

Model 3 adjusted for Model 2 variables plus baPWV at baseline.

*MAFLD* metabolic dysfunction–associated fatty liver disease, *HR* hazard ratio, *CI* confidence interval.

*Rate per 100,000 person-years.

**Table 4:** Subgroup analysis of the interaction between incident arterial stiffness and MAFLD transition patterns in the population free of arterial stiffness at baseline (n = 5656).

Subgroups	MAFLD progression categories							*P* for interaction
	None (n = 2642)	Developed (n = 975)		Regressed (n = 342)		Persistent (n = 1697)		
	HR (95% CI)	HR (95% CI)	*P* value	HR (95% CI)	*P* value	HR (95% CI)	*P* value	
Age								0.671
< 50 years (n = 4274)	1.00 (Reference)	1.06 (0.81–1.39)	0.685	0.96 (0.62–1.49)	0.845	1.35 (1.01–1.81)	0.045	
≥ 50 years (n = 1382)	1.00 (Reference)	1.25 (0.85–1.84)	0.260	0.87 (0.53–1.44)	0.596	1.27 (0.86–1.87)	0.233	
Sex								0.215
Female (n = 1939)	1.00 (Reference)	1.65 (1.14–2.39)	0.009	0.76 (0.35–1.63)	0.478	1.68 (1.06–2.66)	0.028	
Male (n = 3717)	1.00 (Reference)	0.93 (0.71–1.23)	0.629	0.84 (0.58–1.21)	0.351	1.15 (0.88–1.51)	0.317	
Overweight/obesity								0.278
No (n = 2653)	1.00 (Reference)	1.04 (0.77–1.42)	0.801	0.96 (0.50–1.80)	0.895	1.58 (1.04–2.40)	0.033	
Yes (n = 3003)	1.00 (Reference)	1.14 (0.72–1.82)	0.580	0.74 (0.48–1.13)	0.160	1.31 (0.94–1.83)	0.108	
Hypertension								0.288
No (n = 5207)	1.00 (Reference)	1.22 (0.96–1.54)	0.105	0.76 (0.52–1.10)	0.143	1.29 (0.99–1.66)	0.055	
Yes (n = 449)	1.00 (Reference)	1.12 (0.58–2.18)	0.734	1.16 (0.57–2.39)	0.682	1.29 (0.68–2.43)	0.438	
Diabetes								0.255
No (n = 5407)	1.00 (Reference)	1.16 (0.92–1.44)	0.204	0.90 (0.65–1.26)	0.544	1.29 (1.02–1.64)	0.037	
Yes (n = 249)	1.00 (Reference)	0.86 (0.20–3.66)	0.836	0.76 (0.14–4.18)	0.747	2.99 (0.90–9.93)	0.074	
Dyslipidemia								0.937
No (n = 3922)	1.00 (Reference)	1.15 (0.89–1.49)	0.284	0.92 (0.62–1.36)	0.665	1.39 (1.05–1.85)	0.022	
Yes (n = 1734)	1.00 (Reference)	1.14 (0.72–1.82)	0.580	0.75 (0.41–1.37)	0.349	1.16 (0.74–1.81)	0.515	

Estimated from Cox proportional hazard models.

The multivariable model was adjusted as in Model 3 of Table 3.

*MAFLD* metabolic dysfunction–associated fatty liver disease, *HR* hazard ratio, *CI* confidence interval.

To gain more insight into the effects of cardiometabolic risk factor changes on incident arterial stiffness in the MAFLD population, Cox regression analyses were also fitted within persistent MAFLD participants without arterial stiffness at baseline (n = 1697) (Table 5). The fasting glucose, triglyceride, systolic blood pressure and diastolic blood pressure change rates were observed to be significantly associated with incident arterial stiffness, and the effects were attributed mostly to the elevated levels of glucose and triglycerides. Meanwhile, the age-adjusted HR (95% CI) for arterial stiffness development comparing participants with a high probability of fibrosis to those with a low probability of fibrosis in the persistent MAFLD population was 1.92 (1.21–3.04; p = 0.005; Supplemental Table S2). The association persisted after further adjustment for related confounder variables (Model 2) but disappeared after adjustment for baseline ba-PWV (Model 3).

**Table 5:** Associations between the change rate of metabolic risk factors and incident arterial stiffness in the persistent MAFLD population (n = 1697).

Risk factor (Model)	HR (95% CI)	*P* value
Multivariable + BMI change rate (kg/m^2^/year)	1.39 (0.96–2.02)	0.083
Multivariable + WC change rate (cm/year)	0.98 (0.89–1.09)	0.770
Multivariable + SBP change rate (mmHg/year)	1.17 (1.14–1.19)	< 0.001
Multivariable + DBP change rate (mmHg/year)	1.22 (1.18–1.26)	< 0.001
Multivariable + FBG change rate (mmol/l/year)	1.61 (1.22–2.12)	0.001
multivariable + Triglycerides change rate (mmol/l/year)	1.26 (1.12–1.42)	< 0.001
multivariable + HDL-C change rate (mmol/l/year)	0.58 (0.06–5.46)	0.636
Multivariable + above seven factors		
BMI change rate (kg/m^2^/year)	1.12 (0.72–1.74)	0.629
WC change rate (cm/year)	0.94 (0.84–1.06)	0.307
SBP change rate (mmHg/year)	1.13 (1.09–1.18)	< 0.001
DBP change rate (mmHg/year)	1.07 (1.01–1.13)	0.017
FBG change rate (mmol/l/year)	1.45 (1.10–1.90)	0.007
Triglycerides change rate (mmol/l/year)	1.22 (1.06–1.39)	0.004
HDL-C change rate (mmol/l/year)	0.61 (0.07–5.12)	0.647

Estimated from Cox proportional hazard models.

The change rate of metabolic risk factors calculated by the difference between two visits divided by the time interval (years).

The multivariable model was adjusted for age, sex, year of screening exam, education level, current smoking, current drinking, physical activity, anti-hypertensive medication, anti-diabetes mellitus medication, lipid-lowing medication, FIB–4 and estimated glomerular filtration rate and baPWV at baseline. Metabolic indexes, including waist circumference, systolic blood pressure, fasting glucose, triglycerides, LDL cholesterol, and HDL cholesterol, were not adjusted in the analyses because they represent an intrinsic difference in the change rate.

*CI* confidence interval, *HR* hazard ratio.

### Sensitivity analyses

We used the higher value instead of the mean value of the right and left ba-PWV and repeated the main analysis. Although the results became statistically nonsignificant, this did not generally affect the trend of these associations (Supplemental Tables S3 and S4).

## Discussion

This large cohort study was conducted to examine whether dynamic changes in MAFLD status were associated with the progression of arterial stiffness. To our knowledge, the present study is one of the few studies on the relationship between MAFLD dynamic change and subclinical arteriosclerosis in the Chinese population. We confirmed that persistent MAFLD was significantly associated with an increased risk of developing arterial stiffness. Of note, the effect was robust after adjustment for potentially cardiometabolic risks and baseline ba-PWV. Furthermore, our study found that the risk was more prominent in the specific persistent MAFLD subgroup with higher increased levels of glucose and triglycerides. These findings suggest that improving MAFLD status may yield benefits related to cardiovascular health.

CVD is the most common cause of death in individuals with MAFLD, and subclinical arteriosclerosis is an early stage of CVD. Exploring the relationship between MAFLD and subclinical arteriosclerosis is extremely important for early prevention and health promotion. A growing body of evidence has demonstrated that MAFLD not only behaves as a marker of CVD but also might take part in its pathogenesis, providing insight regarding the relationship between MAFLD and early-stage atherosclerosis^29–31^. Although several studies have previously evaluated the independent correlation between MAFLD and individual indices of subclinical atherosclerosis, most of the studies were observational rather than prospective^32^. The cross-sectional design fails to explore causal or temporal associations between MAFLD and the development of subclinical atherosclerosis. Additionally, a single point assessment of subclinical atherosclerosis and MAFLD status was accounted for these studies, while dynamic assessments at more time points could reflect progression and provide more useful information. Moreover, the sample of the majority of studies was hospital based, so selection bias was inevitable and might lead to an overestimation of the relationship.

Some reports have shown that MAFLD correlates more strongly with CVD than NAFLD^11,13,14^. However, there are few studies on the relationship between MAFLD and subclinical atherosclerosis at present. Bessho et al. conducted a cross-sectional study including 890 Japanese health check-up patients and found that MAFLD at baseline was significantly associated with the subclinical atherosclerosis indices ba-PWV, CAC and hs-CRP^20^. Liu et al. conducted a Chinese community-based cohort study including 6232 participants aged 40 years or older and found that MAFLD was significantly associated with higher risks of developing subclinical atherosclerosis, as assessed by CIMT, ba-PWV and microalbuminuria^33^. Our study was based on a prospective cohort including a larger number of health check-up individuals and confirmed that persistent MAFLD was significantly associated with arterial stiffness assessed by ba-PWV compared to non-MAFLD. These findings highlight the importance of screening subclinical atherosclerosis in the MAFLD population for early intervention and prevention of CVD.

Measurement of indicators such as weight, metabolic status, arteriosclerosis, etc., at an initial or a single time point while ignoring potential change patterns over time may underestimate health risks. An increasing number of studies have focused on the dynamic change trajectories of various indicators for the prediction of disease risks. In another study, we found that the effect of NAFLD at baseline on the deterioration of metabolically healthy status was stronger in the “fluctuating obese” phenotype change pattern group than in the “stable obese” phenotype change pattern group^24,34^. Moreover, Sinn et al. revealed that persistent NAFLD was associated with an increased risk of subclinical carotid atherosclerosis development in a retrospective cohort study of 8020 adult men (average age: 49.2 years)^17^. However, there are few studies on the relationship between MAFLD dynamic changes and subclinical atherosclerosis. Liu et al. recently conducted a community-based cohort study including 6232 participants aged 40 years or older in Shanghai, China, with a median of 4.3 years of follow-up and reported that participants with stable MAFLD showed 17.6%, 32.4%, and 35.4% increased risks of developing elevated CIMT, elevated ba-PWV and microalbuminuria compared with those with stable non-MAFLD^33^. Similarly, we explored the relationship between the MAFLD change patterns and ba-PWV and found that the association was most obvious in the persistent MAFLD group, followed by the developed MAFLD group when the none MAFLD group was used as a reference. Our study further provides important evidence of the risks of incident subclinical atherosclerosis in association with MAFLD transitions, which highlights the importance of early interventions for individuals with persistent MAFLD, even leading to reversion, to prevent clinical atherosclerosis.

The biological mechanisms underlying the correlation between MAFLD and subclinical atherosclerosis remain to be elucidated. MAFLD and atherosclerosis frequently coexist because they share pathogenic mechanisms. NASH and atherosclerosis were suggested as two aspects of a shared disease with a common etiology involving metabolic and inflammatory factors^35–38^. Accumulating evidence suggests that MAFLD is not merely affected by insulin resistance but could also act as a stimulus for further insulin resistance and metabolic syndrome in turn, thus paving the way for the development and progression of atherosclerosis and overt CVD events^36^. With fat accumulation, oxidative stress, macrophage activation, and endothelial dysfunction, MAFLD could accelerate the development of atherosclerosis through common pathways of chronic low-grade inflammation and adipokine imbalance^39–43^. Further prospective studies are needed to establish whether a causative relationship exists between MAFLD and subclinical atherosclerosis progression and whether individuals with MAFLD may benefit from early evaluation of atherosclerosis, thus facilitating prediction of CVD morbidity.

Notably, annual change rates of fasting glucose, triglycerides and blood pressure were significantly associated with incident arterial stiffness in the MAFLD population, and fasting glucose had the highest effect. Similarly, the following research drew similar conclusions to those of our research. Matsubayashi et al. found that there was a significantly increased risk of CVD only in MAFLD participants with metabolic syndrome^19^. Yoneda et al. revealed that the concurrent prevalence of diabetes and hypertriglyceridemia among NAFLD/MAFLD patients was high and may affect the development of CVD^21^. Furthermore, Bessho et al. reported that diabetes-MAFLD could be a significant risk factor for cardiovascular disease through insulin resistance and low-grade inflammation^20^. Lee et al. found that participants with diabetes-MAFLD had a higher cardiovascular disease risk than those with overweight-MAFLD and lean-MAFLD^18^. These studies focused on CVD, not subclinical atherosclerosis, which differed from our study. In summary, these findings suggest that the MAFLD population should be reclassified by the status of high glucose, high blood pressure and other metabolic indicators to identify high-risk subpopulations and facilitate precise treatment and management. Furthermore, clinicians should pay close attention to MAFLD subjects with annually increasing fasting glucose, triglycerides and blood pressure as being at high risk for cardiovascular disease^44^.

Several studies have reported that a high probability of fibrosis in the MAFLD population could trigger the progression of subclinical arteriosclerosis and CVD^45,46^. Liu et al. found that MAFLD regressed to non-MAFLD with a low probability of fibrosis group had a significant decrease in ba-PWV when using the stable MAFLD group as a reference. They concluded that the regression of MAFLD might modify the risks of developing subclinical atherosclerosis, especially among those with a low probability of fibrosis. Our study found that the age-adjusted HR for arterial stiffness development comparing participants with a high probability of fibrosis to those with a low probability of fibrosis in the persistent MAFLD population was 1.92 (1.21–3.04; p = 0.005). The association persisted after further adjustment for related confounder variables (Model 2) but disappeared after adjustment for baseline ba-PWV (Model 3). However, the sample of persistent MAFLD with a high probability of fibrosis was small (n = 38), and the results should be interpreted cautiously. Future prospective studies with larger sample sizes and longer follow-up periods are needed to further demonstrate the results.

To our knowledge, this study was one of the few studies to investigate the associations of MAFLD dynamic change (not a single-point status) with the development of arterial stiffness in a large health check-up population in China, filling a gap in the understanding of the effect of MAFLD transitions on the risks of subclinical atherosclerosis. In addition, we had rich data on health behaviors and sociodemographic information; thus, we were able to examine the relationship after controlling for a comprehensive set of confounders. However, several limitations were identified. First, despite the large cohort size, this was not a community-based study but was rather a health checkup-based cohort study. Our subjects were not necessarily representative of the general Chinese or Asian population, so our findings should be confirmed by a multicenter cohort study. Second, although we used two dimensions of arterial stiffness (the annual ba-PWV increase and incident arterial stiffness), other subclinical atherosclerosis markers, such as carotid plaques, intima-media thickness (CIMT), CAC and FMD, were not included. Third, liver fibrosis is associated with the development of subclinical carotid atherosclerosis, and we used noninvasive tests of the FIB-4 score as a confounder for liver fibrosis, but histological data to identify the severity of liver disease were absent^17^. Fourth, although ultrasonography is widely (applied in 90.56% of all NAFLD-related studies in China) and accurately (pooled sensitivity, 84.8%; specificity, 93.6%) performed to detect fatty liver, we did not use biopsy to identify the severity of NAFLD^25^. We applied noninvasive biochemistry markers, such as FIB-4, ALT, albumin and total bilirubin levels, for calibration. Fifth, fasting insulin or C-reactive protein levels were not available in this study. These missing data might cause some MAFLD participants to be misclassified as non-MAFLD, leading to an underestimation of the association between MAFLD and the progression of arterial stiffness.

## Conclusions

Persistent MAFLD was associated with a higher risk of arterial stiffness development in a Chinese health check-up population. The association was different in regard to change rates of cardiometabolic risk factors, suggesting that the persistent MAFLD population should be reclassified by the status of metabolic indicator changes to identify high-risk subpopulations and facilitate precise treatment and management.

## Supplementary Information

Supplementary Information.

## Data Availability

The datasets used and analyzed during the current study are available from the corresponding author on reasonable request. Restrictions apply according to national and international regulations for personal data protection.
