# Supplementary material for: Transition patterns of metabolic dysfunction-associated fatty liver disease status in relation to arterial stiffness progression: a health check-up cohort study
Source: Sci Rep. 2023 Jun 15;13:9690. doi: 10.1038/s41598-023-35733-0 (PMC10272131; doi:10.1038/s41598-023-35733-0)
Supplement: Supplementary file 1 — Supplementary Information. [file 41598_2023_35733_MOESM1_ESM.pdf]

Transition patterns of metabolic dysfunction-associated fatty liver disease status in relation to arterial stiffness progression: A health check-up cohort study

Clinical and lifestyle characteristics definition

Normal weight (18.5 kg/m^2 ≤ BMI <24 kg/m^2), overweight (24 kg/m^2 ≤ BMI <28 kg/m^2), and obesity (BMI ≥28 kg/m^2) were defined using the standard weight criteria for adults in China. Hypertension was defined as systolic blood pressure ≥140 mm Hg or diastolic blood pressure ≥ 90 mm Hg or specific drug treatment[1]. Type 2 diabetes was diagnosed by fasting glucose ≥ 126 mg/dL or specific drug treatment[2]. Dyslipidemia was defined as the presence of low-density lipoprotein cholesterol (≥ 140 mg/dl), high-density lipoprotein cholesterol (≥ 40 mg/dl), elevated triglyceride level (≥ 150 mg/dl), or treatment for dyslipidemia[3].

Current smoking was coded 'present' if more than one cigarette per day (on average) was consumed over a period longer than six months. Current drinking was coded 'present' if the individual reported consumption of beer, wine (including Chinese wine) and/or liquor at least two days per week over a period exceeding 12 months. Individuals were considered physically active when reporting more than three days of moderate-intensity exercise.

Positive liver ultrasound findings included the following 5 criteria: 1) parenchymal brightness, 2) liver-to-kidney contrast, 3) deep beam attenuation, 4) bright vessel walls, and 5) gallbladder wall definition[4]. Subjects with at least two abnormal findings were diagnosed with hepatic steatosis[5].

References

[1] P.A. James, S. Oparil, B.L. Carter, et al. 2014 evidence-based guideline for the management of high blood pressure in adults: report from the panel members appointed to the Eighth Joint National Committee (JNC 8)[J]. JAMA. 2014, 311(5), 507-520. DOI: 10.1001/jama.2013.284427.

[2] W. Kerner, J. Bruckel, A. German Diabetes Definition, classification and diagnosis of diabetes mellitus[J]. Exp. Clin. Endocrinol. Diabetes. 2014, 122(7), 384-386. DOI: 10.1055/s-0034-1366278.

[3] W. Yang, J. Xiao, Z. Yang, et al. Serum lipids and lipoproteins in Chinese men and women[J]. Circulation. 2012, 125(18), 2212-2221. DOI: 10.1161/CIRCULATIONAHA.111.065904.

[4] N.N. III. Hepatic Steatosis Ultrasound Images Assessment Procedures Manual 2010.[J].

[5] G.C. Farrell, S. Chitturi, G.K. Lau, et al. Guidelines for the assessment and management of non-alcoholic fatty liver disease in the Asia-Pacific region: executive summary[J]. J. Gastroenterol. Hepatol. 2007, 22(6), 775-777. DOI: 10.1111/j.1440-1746.2007.05002.x.

Supplemental Table 1. Study population clinical characteristics at baseline (n = 8,807) stratified by MAFLD status.

	MAFLD	
	**No**	**Yes**
**Prevalence, n (%)**	5273 (59.9)	3534 (40.1)
**Demographic factors**		
Age, years	46.6 ± 10.1	46.8 ± 9.2
Male sex, n (%)	3067 (58.2)	3025 (85.6) [a]
University degree, n (%)	2400 (45.5)	1724 (48.8) [a]
**Lifestyle status**		
Current smoker, n (%)	1025 (19.4)	908 (25.7) [a]
Current drinker, n (%)	1492 (28.3)	1257 (35.6) [a]
Physical activity, n (%)	2119 (40.2)	1203 (34.0) [a]
**Classic vascular risk factors**		
Body-mass index, kg/m^2	23.3 ± 2.8	26.6 ± 2.5 [a]
Waist circumference, cm	80.2 ± 8.6	90.4 ± 7.0 [a]
Heart rate, beats/min	71.2 ± 10.9	72.5 ± 10.7 [a]
Systolic blood pressure, mm Hg	122.3 ± 16.4	127.4 ± 14.6 [a]
Diastolic blood pressure, mm Hg	76.1 ± 11.3	81.3 ± 11.0 [a]
Hypertension, n (%)	767 (14.5)	847 (24.0) [a]
Anti-hypertensive medication, n (%)	288 (5.5)	383 (7.6) [a]
Fasting glucose, mmol/L	5.31 ± 1.14	5.66 ± 1.36 [a]
Diabetes mellitus, n (%)	254 (4.8)	392 (11.1) [a]
Anti-diabetes medication, n (%)	92 (1.7)	196 (5.5) [a]
Triglycerides, mmol/L	1.26 (0.91, 1.84)	2.02 (1.41, 3.03) [a]
HDL–C cholesterol, mmol/L	1.51 ± 0.38	1.29 ± 0.27 [a]
LDL–C cholesterol, mmol/L	2.77± 0.83	2.77 ± 0.88
Dyslipidemia, n (%)	1334 (25.3)	1771 (50.1) [a]

Anti-dyslipidemia medication, n (%)	86 (1.6)	61 (1.7)
**Emerging risk factors and others**		
Albumin, g/L	46.1 ± 2.7	46.7 ± 2.7 [a]
Total bilirubin, µmol/L	15.5 ± 5.1	15.3 ± 5.3
ALT, U/L	20.0 (15.0, 28.0)	30.0 (22.0, 43.0) [a]
AST, U/L	22.0 (18.0, 25.0)	24.0 (21.0, 28.0) [a]
FIB–4	0.47 (0.33, 0.66)	0.36 (0.25, 0.51) [a]
eGFR, mL/min/1.73m^2	109.9 (95.4, 126.1)	105.1 (92.3, 120.2) [a]
**Arterial stiffness**		
Ba-PWV, cm/s	1300 (1181, 1451)	1365 (1245, 1510) [a]
Increased ba-PWV, n (%)	1656 (31.4)	1495 (42.3) [a]

HDL, high-density lipoprotein; LDL, low-density lipoprotein; ALT, alanine aminotransferase; AST: aspartate aminotransferase; eGFR, estimated glomerular filtration rate; ba-PWV, brachial–ankle pulse wave velocity.

Supplemental Table 2. The fibrosis probability of MAFLD at baseline with incident arterial stiffness in the persistent MAFLD population (n = 1,697).

	Person-years	Incident cases	Rate*	The incident arterial stiffness					
				Model 1		Model 2		Model 3	
				HR (95% CI)	*P* value	HR (95% CI)	*P* value	HR (95% CI)	*P* value
FIB-4 score									
Low (< 1.30) (n = 1659)	7344.8	479	65.22	1.00 (Reference)		1.00 (Reference)		1.00 (Reference)	
High (≥1.30) (n = 38)	175.1	19	108.52	1.92 (1.21–3.04)	0.005	1.82 (1.02–3.32)	0.042	1.65 (0.92–2.96)	0.095

Estimated from Cox proportional hazard models.
*Rate per 100,000 person-years.
Model 1 adjusted for sex. Age-adjusted models were not calculated because age was used for calculating the FIB-4 scores.
Model 2 adjusted for Model 1 variables plus year of screening exam, education level, current smoking, current drinking, physical activity, anti-hypertensive medication, anti-diabetes mellitus medication, anti-dyslipidemic medication, waist circumference, systolic blood pressure, fasting glucose, triglycerides, LDL cholesterol, HDL cholesterol, estimated GFR, total bilirubin and albumin at baseline.

Model 3 adjusted for Model 2 variables plus ba-PWV at baseline.
Abbreviations: MAFLD, metabolic dysfunction–associated fatty liver disease; HR, hazard ratio; CI, confidence interval.

Supplemental Table 3. Association between MAFLD transition patterns and the annual increase in ba-PWV in all population samples (n = 8,807).

	The annual ba-PWV change rate					
	Model 1		Model 2		Model 3	
	coefficient β (95% CI)	*P* value	coefficient β (95% CI)	*P* value	coefficient β (95% CI)	*P* value
MAFLD progression status						
None (n = 3674)	1.00 (Reference)		1.00 (Reference)		1.00 (Reference)	
Developed (n = 1599)	7.50 (2.55–12.46)	0.003	7.24 (1.39–13.10)	0.015	6.77 (4.14–9.40)	<0.001
Regressed (n = 592)	-2.75 (-10.06–4.56)	0.461	-1.15 (-9.06–6.77)	0.777	1.32 (-2.23–4.86)	0.466
Persistent (n = 2942)	6.14 (1.88–10.41)	0.005	8.96 (3.26–14.66)	0.002	6.86 (4.16–9.56)	<0.001

Estimated from linear mixed models.
Model 1 adjusted for age and sex.
Model 2 adjusted for Model 1 variables plus education level, current smoking, current drinking, physical activity, presence of hypertension and diabetes mellitus, lipid-lowing medical use, heart rate, waist circumference, systolic blood pressure, fasting glucose, triglycerides, LDL cholesterol, HDL cholesterol, FIB–4, estimated GFR, total bilirubin and albumin at baseline.
Model 3 adjusted for Model 2 variables plus ba-PWV at baseline.
MAFLD, metabolic dysfunction–associated fatty liver disease; CI, confidence interval.

Supplemental Table 4. Association between MAFLD transition patterns and incident arterial stiffness in the population free of arterial stiffness at baseline (n = 5,253).

	Person-years	Incident cases	Rate*	The incident arterial stiffness					
				Model 1		Model 2		Model 3	
				HR (95% CI)	*P* value	HR (95% CI)	*P* value	HR (95% CI)	*P* value
None (n = 2519)	10087.6	424	42.0	1.00 (Reference)		1.00 (Reference)		1.00 (Reference)	

Developed (n = 905)	4142.3	288	69.5	1.44 (1.24–1.69)	<0.001	1.30 (1.05–1.61)	0.018	1.20 (0.97–1.49)	0.159
Regressed (n = 318)	1485.0	84	56.6	0.89 (0.70–1.14)	0.350	0.99 (0.73–1.36)	0.980	0.95 (0.70–1.30)	0.386
Persistent (n = 1511)	6700.3	467	69.7	1.43 (1.24–1.64)	<0.001	1.35 (1.07–1.72)	0.012	1.25 (0.98–1.58)	0.064

Estimated from Cox proportional hazard models.
*Rate per 100,000 person-years.
Model 1 adjusted for age and sex.
Model 2 adjusted for Model 1 variables plus year of screening exam, education level, current smoking, current drinking, physical activity, anti-hypertensive medication, anti-diabetes mellitus medication, anti-dyslipidemic medication, waist circumference, systolic blood pressure, fasting glucose, triglycerides, LDL cholesterol, HDL cholesterol, FIB–4, estimated GFR, total bilirubin and albumin at baseline.
Model 3 adjusted for Model 2 variables plus ba-PWV at baseline.

Abbreviations: MAFLD, metabolic dysfunction–associated fatty liver disease; HR, hazard ratio; CI, confidence interval.
